# CD4+ T cells in aged or thymectomized recipients of allogeneic stem cell transplantations

**DOI:** 10.1186/s40659-015-0033-8

**Published:** 2015-07-26

**Authors:** Hiroshi Takahashi, Kazuhiko Ikeda, Kazuei Ogawa, Syunnichi Saito, Alain M Ngoma, Yumiko Mashimo, Koki Ueda, Miki Furukawa, Akiko Shichishima-Nakamura, Hiroshi Ohkawara, Kenneth E Nollet, Hitoshi Ohto, Yasuchika Takeishi

**Affiliations:** Department of Cardiology and Hematology, School of Medicine, Fukushima Medical University, Fukushima, Japan; Department of Blood Transfusion and Transplantation Immunology, School of Medicine, Fukushima Medical University, 1 Hikariga-oka, Fukushima, Fukushima 960-1295 Japan; Department of Epidemiology, Biostatistics and Occupational Health, McGill University, Montreal, QC Canada

**Keywords:** Regulatory T cells, Allogeneic hematopoietic stem cell transplantation, Thymus, Graft-versus-host disease

## Abstract

**Background:**

CD4+CD25highFOXP3+ regulatory T (Treg) cells, which include thymus-derived and peripherally induced cells, play a central role in immune regulation, and are therefore crucial to prevent graft-versus-host disease (GVHD). The increasing use of allogeneic hematopoietic stem cell transplantation (allo-HSCT) for elderly patients with thymus regression, and our case of allo-HSCT shortly after total thymectomy, raised questions about the activity of thymus-derived Treg cells and peripherally induced Treg cells, which are otherwise indistinguishable.

**Results:**

We found that despite pre-transplant thymectomy or older age, both naïve and effector Treg cells, as well as naïve and effector conventional T cells, proliferated in allo-HSCT recipients. Higher proportions of total Treg cells 1 month post allo-HSCT, and naïve Treg cells 1 year post allo-HSCT, appeared in patients achieving complete chimera without developing significant chronic GVHD, including our thymectomized patient, compared with patients who developed chronic GVHD.

**Conclusions:**

Treg cells that modulate human allogeneic immunity may arise peripherally as well as in the thymus of allo-HSCT recipients.

## Background

Allogeneic hematopoietic stem cell transplantation (allo-HSCT), including bone marrow (BM) transplantation (BMT), peripheral blood (PB) stem cell transplantation (PBSCT), and cord blood transplantation, is widely used for the treatment of malignant and nonmalignant hematologic diseases [1]. However, allo-HSCT often shows comorbidity or mortality due to graft-versus-host disease (GVHD), opportunistic infections, regimen-related toxicity and relapse of the original hematologic disease. Donor immune cells provide graft-versus-leukemia effects and protect against opportunistic infections. However, in GVHD, donor immune cells respond inappropriately to host antigens in a pro-inflammatory background of tissue damage and cytokine dysregulation [2].

Patients with acute GVHD (aGVHD) and chronic GVHD (cGVHD) show deficits of CD4+CD25high regulatory T (Treg) cells, which express the transcription factor, fork-head box P3 (FOXP3), and play a crucial role in control of autoimmunity [2, 3]. Treg cells usually derive from the thymus with upregulation of FOXP3. Thymus also provides naïve conventional T (Tcon) cells, which are considered to be crucial to maintain proper immune responses [4]. Therefore, therapeutic strategies that facilitate recovery of thymopoiesis have been attempted [5]. In addition, there is a second subset of Treg cells, periphery-derived induced Treg cells (iTreg cells), which can be transformed from Tcon cells through upregulation of FOXP3 in the context of TGF-β and IL-2 [6–8]. It has also been reported that alloantigen can induce Treg cells via a thymus-independent pathway in mice [9]. However, the role of iTreg cells in allo-HSCT is unknown because there is no known marker to discriminate iTreg cells from thymus-derived Treg cells.

After the introduction of reduced-intensity conditioning regimens, allo-HSCT increased for elderly patients, for whom the thymus is naturally regressed. In fact, delayed recovery of thymopoiesis, which can be inferred by T cell receptor rearrangement excision circle levels and naïve T cells, has been reported among elderly allo-HSCT recipients [4]. Other investigators showed a more rapid reconstitution of effector T cells in allo-HSCT recipients older than 42 years compared with younger recipients [10]. In addition, even in patients with delayed recovery of thymopoiesis, clinical consequences of allo-HSCT vary. Moreover, we recently encountered a case in which allo-HSCT for acute lymphoblastic leukemia shortly after thymectomy did not provoke significant GVHD. These facts prompted us to investigate the role of thymopoiesis after allo-HSCT, especially in relation to Treg cells. Hence, in this study, we investigated Treg and Tcon cells, including naïve and effector in allo-HSCT recipients in the context of patient age and cGVHD.

## Results

### Allo-HSCT in young and old patients

We investigated 22 patients (Table 1), comparing clinical findings and T cell populations between 14 young (<50 years) and 8 old (≥50 years) recipients. Old recipients more frequently received reduced-intensity conditionings compared with young recipients [6/8 (75%) vs. 2/14 (15%), *P* = 0.038]. There was a trend that PBSCT was more common among old recipients (BMT vs. PBSCT, 3 vs. 5) compared with young recipients (11 vs. 3) (*P* = 0.054). The thymectomized patient received PBSCT from an HLA allele fully matched related donor (30 year-old daughter) after reduced-intensity conditioning for acute lymphoblastic leukemia at complete remission. Her leukemia remains in complete remission at the time of evaluation, with 100% donor-derived cells in PB mononuclear cells (MNCs) and CD3+ cells, and BM MNCs. PB MNCs of all other patients were also in complete chimera (>90% cells were donor-derived).

**Table 1:** Patient characteristics

Variable	Number of patients
Age [median (range), years]	38.5 (23–61)
Male/female	14/8
Diagnosis	
AML/MDS	14
ALL/ML	8
Donor	
Related	10
Unrelated	12
Source	
BMT	14
PBSCT	8
HLA compatibility	
Matched	17
1-locus mismatch	5
Conditioning	
Myeloablative	14
TBI/CY-based	10
BU/CY	4
Reduced-intensity	8
TBI/Flu/BU	4
TBI/Flu/Mel	4
GVHD prophylaxis	
CyA/MTX	3
TAC/MTX	19

*AML/MDS* acute myeloid leukemia/myelodysplastic syndromes, *ALL/ML* acute lymphoblastic leukemia/malignant lymphoma, *BMT* bone marrow transplantation, *PBSCT* peripheral blood stem cell transplantation, *HLA* human leukocyte antigen, *TBI* total body irradiation, *CY* cyclophosphamide, *BU* Busulfan, *Mel* melphalan, *GVHD* graft-versus-host disease, *CyA* cyclosporine A, *MTX* methotrexate, *TAC* tacrolimus.

### CD4+ conventional and regulatory T cells in young and old allo-HSCT recipients early after transplantation

**Fig. 1:**
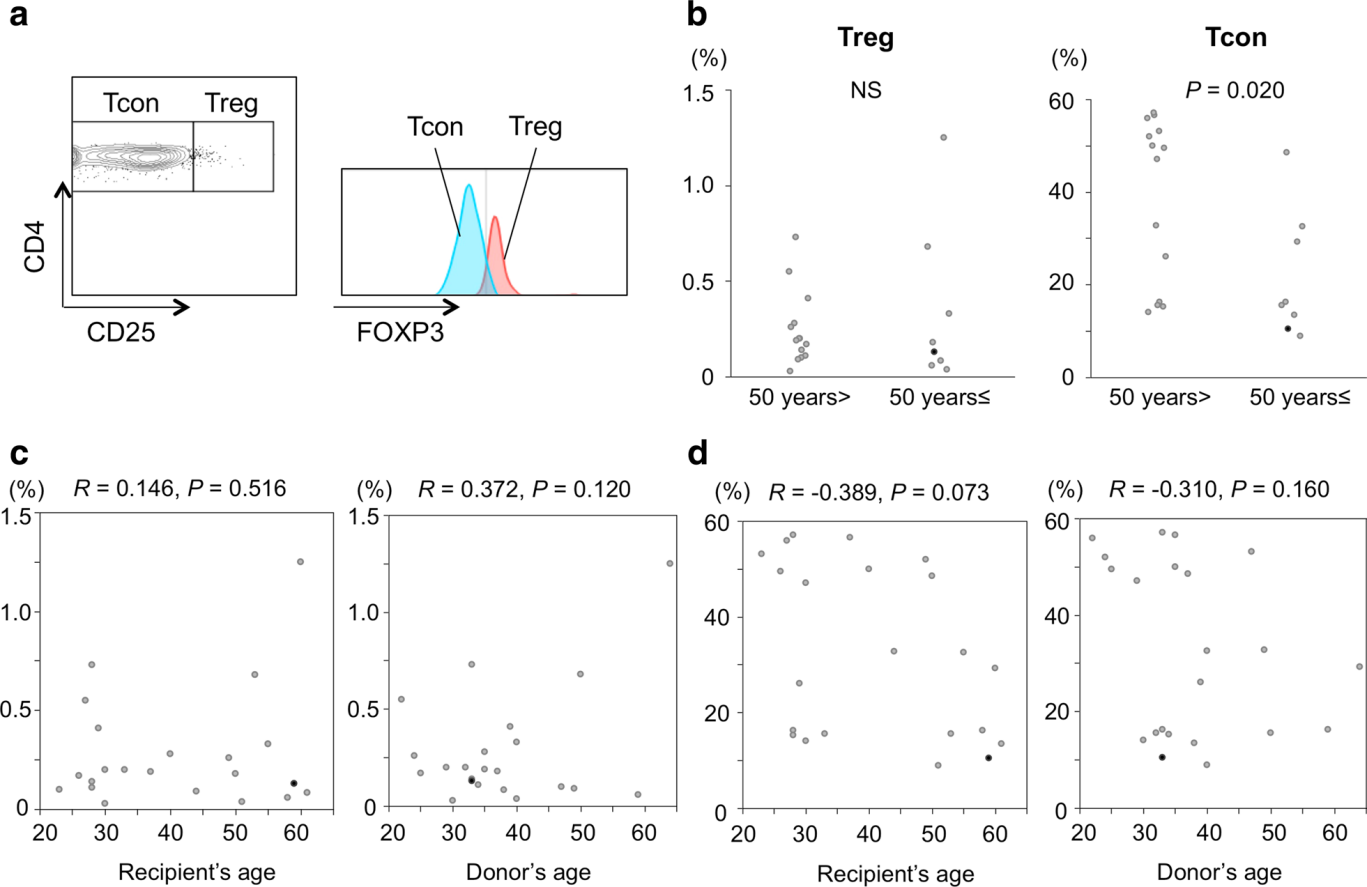
CD4+ regulatory T (Treg) and conventional T (Tcon) cells at 1 month after allo-HSCT. **a** Flow cytometric analysis of the patient who had undergone thymectomy prior to PBSCT. Both CD4+CD25high Treg cells and CD4+ Tcon cells were among the CD4+ cells detected by flow cytometry of peripheral blood mononuclear cells at 1 month after PBSCT. Treg cells expressed Foxp3 more abundantly than Tcon cells. **b** Comparisons in the proportions of CD4+CD25highFoxp3+ Treg cells and CD4+Foxp3− Tcon cells between young and old allo-HSCT recipients. Correlations between proportions of Treg cells (**c**) or Tcon cells (**d**) and ages of recipients or donors. The *close circles* indicate data of the thymectomized patient.

**Fig. 2:**
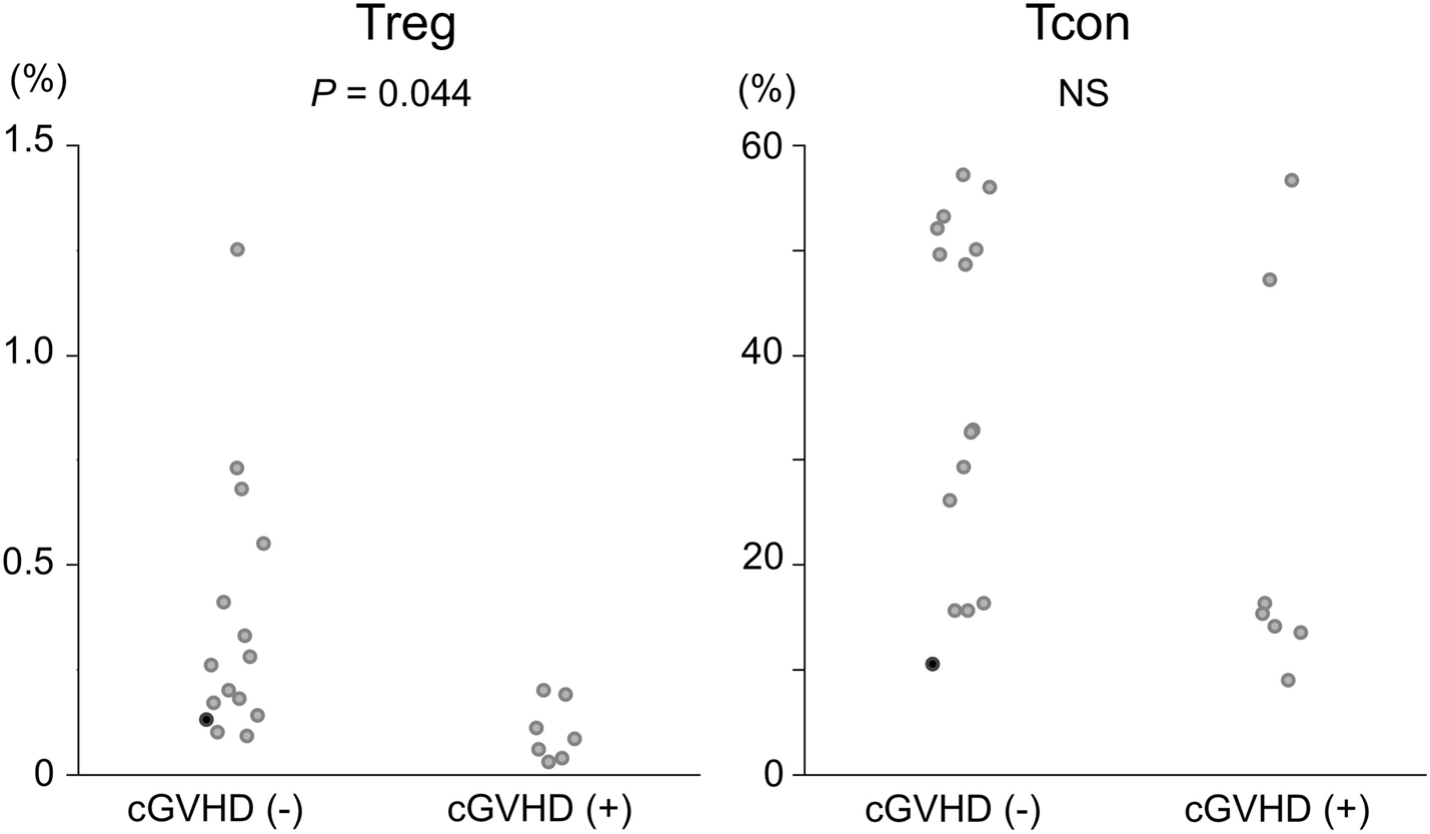
Comparisons of Treg and Tcon proportions between allo-HSCT recipients who eventually developed cGVHD and those without cGVHD. The *close circles* indicate data of the thymectomized patient.

At day 30 after allo-HSCT, we performed 3-color flow cytometry, in which CD4+CD25highFoxp3+ lymphocytes and all other CD4+Foxp3− lymphocytes were defined as Treg cells and Tcon cells, respectively (Fig. 1a) [11]. Proportions of Tcon cells, rather than Treg cells, were significantly greater in young recipients compared with old recipients 1 month after allo-HSCT (Fig. 1b). Proportions of Treg cells were not correlated with ages of either recipients or donors (Fig. 1c), whereas there was a trend (*P* = 0.073) that proportions of Tcon cells were negatively correlated with recipient’s age, but not donor’s (Fig. 1d). On the other hand, we found that proportions of Treg cells were already lower at 1 month after allo-HSCT in patients who eventually developed clinically significant cGVHD compared with those without (Fig. 2). There were no significant differences in proportions of Treg cells (0.24 ± 0.20 vs. 0.36 ± 0.42%) and Tcon cells (38 ± 19 vs. 23 ± 13%) between BMT recipients and PBSCT recipients. At this point, there were not any differences in the absolute lymphocyte counts between young recipients and old recipients (0.59 ± 0.47 vs. 0.56 ± 0.26 × 10^9^/L), and both Treg cells and Tcon cells were already detectable in the thymectomized patient (Fig. 1a), who also had already achieved complete chimera with 100% donor-derived PB MNCs and BM MNCs at this point.

### Naïve and effector T cells in allo-HSCT recipients 1 year after transplantation

**Fig. 3:**
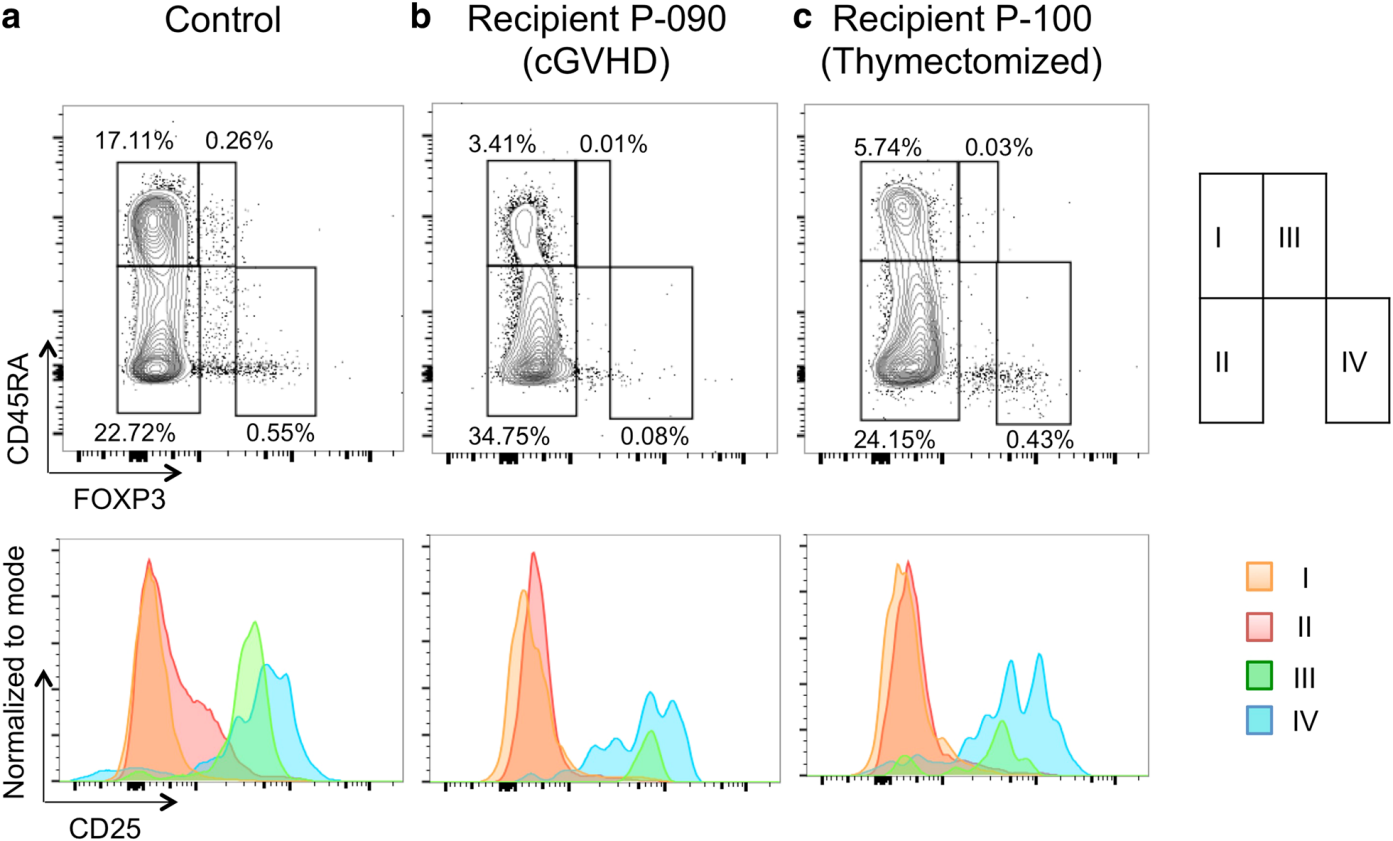
Detection of naïve and effector CD4+ cells in peripheral blood mononuclear cells. According to Ref. [14], naïve Tcon cells (fraction *I*), effector Tcon cells (*II*), naïve Treg cells (*III*), and effector Treg cells (*IV*) were CD4+ by flow cytometry. CD25 expression in each subpopulation is shown in the *histogram*. Representative data from a healthy donor, a patient with chronic GVHD (cGVHD), and our thymectomized patient are shown.

**Fig. 4:**
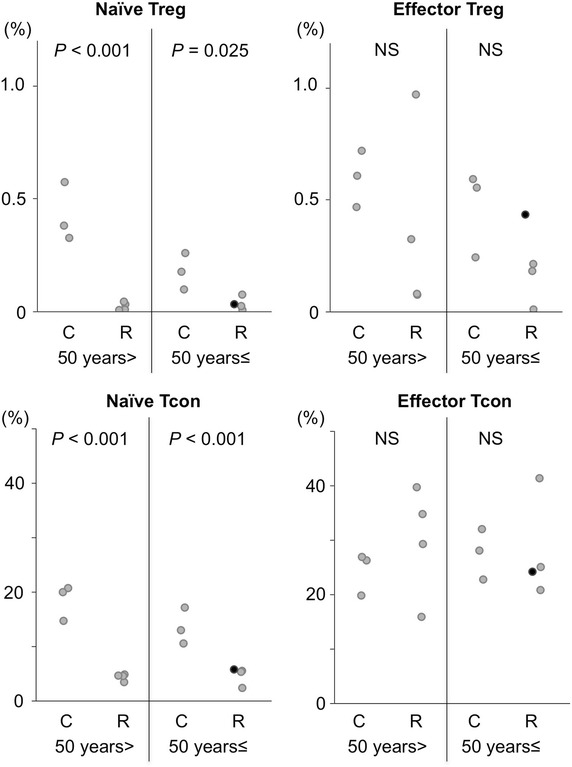
Comparisons in the proportions of naïve and effector Treg cells and Tcon cells between allo-HSCT recipients and healthy controls. Proportions of indicated cells in allo-HSCT recipients (R), determined by flow cytometry as shown in Fig. 3, were compared with age-matched controls (C). The *close circles* indicate data of the thymectomized patient.

We studied proportions of naïve and effector fractions of Treg cells and Tcon cells (Fig. 3) [12], in young and old recipients at approximately 1 year after allo-HSCT. At this point, both in Treg cells and Tcon cells, CD45RA+ naïve cells remained at significantly low proportions in allo-HSCT recipients, regardless of age (Fig. 4). However, these naïve cells, as well as CD45RA− effector cells, were certainly detectable in all of these patients examined, even in the thymectomized patient (Fig. 3c), whose complete chimera still persisted with 100% donor-derived PB MNCs and CD3+ lymphocytes, and BM MNCs at this point. Proportions of both naïve Treg cells and Tcon cells were not different between young and old recipients. We also compared proportions of Treg cells and Tcon cells with respect to cGVHD. In patients with clinically significant cGVHD, we found significantly lower proportions of Treg cells, especially in the naïve fraction (0.015 ± 0.011 vs. 0.049 ± 0.022%, *P* = 0.024), compared with those without. In contrast, proportions of effector Tcon cells were significantly greater in patients with clinically significant cGVHD compared with other patients (34.0 ± 6.9 vs. 20.2 ± 4.2%, *P* = 0.022).

## Discussion

After allo-HSCT, the T-cell compartment is slowly reconstituted with both thymus-independent and -dependent pathways [4]. Early after transplantation, the thymus-independent pathway by either adoptively transferred donor-derived T cells or recipient-derived T cells that survive conditioning treatment predominates. The transferred T cells expand in response to early post-transplant circumstances with lymphopenia and high cytokine levels, and oligoclonal proliferation associated with cognate antigens. Another pathway, which is a more prolonged process of reconstitution of functional T cells with sufficient and broad antigenic specificity, depends on the de novo production of naïve T cells by the thymus. Thus, thymic regeneration may be crucial to supply new Tcon cells and Treg cells that contribute to prevention of relapsing hematologic malignancies, opportunistic infections, and cGVHD [5, 13]. We found a lower frequency of Tcon cells rather than Treg cells early after allo-HSCT in the elderly recipients (Fig. 1). Our present study, however, revealed that naïve and effector Treg cells, as well as naïve and effector Tcon cells, exist even in allo-HSCT recipients more than 50 years old, including our surgically athymic patient, at 1 year after allo-HSCT (Fig. 3). The detailed kinetics of Treg cells is unclear in allo-HSCT recipients, but proportions of naïve Treg cells and Tcon cells were lower in recipients compared with healthy controls, independent of recipient or donor age, at both 1 month and 1 year in the present study. Next, we observed lower frequencies of Treg cells at both 1 month and 1 year after allo-HSCT in patients who eventually developed clinically significant cGVHD, consistent with previous studies [14, 15]. Imanguli et al. [16] recently compared proportions of naïve and effector Treg cells between patients with cGVHD and normal controls with the same method used in our present study [12]. They found significantly lower frequency of naïve Treg cells, but not effector Treg cells, in allo-HSCT recipients compared with controls. In our present study, regardless of the presence of cGVHD, allo-HSCT recipients consistently showed decreased naïve Tregs. Nevertheless, we found that proportions of Treg cells, especially in the naïve fraction, were significantly lower in patients with cGVHD compared with patients without cGVHD, despite a small cohort with heterogeneities including donor sources and conditioning protocols in the present study. Another concern of this study is that the thymectomized patient belongs to the older group. These facts limit the confidence that can be placed in our findings. However, it has been recently reported that impaired reconstitution of naïve Treg cells was also associated with aGVHD [17] as well as cGVHD, lending support to the importance of naïve Tregs in the modulation of allogeneic immune phenomena that cause GVHD.

In patients who were coincidentally thymectomized through cardiac surgery, atopic and autoimmune-like symptoms with elevated anti-dsDNA antibodies were frequently found [18]. Such autoimmune-like diseases were associated not with total numbers of Treg cells, but Treg homeostasis preserved by naïve Treg cells in these post-surgery individuals with impaired thymopoiesis. In an established mouse model, female B6AF1 mice thymectomized on day 3 develop autoimmune ovarian disease and dacryoadenitis [19, 20]. These mice possess disease-relevant Treg cells of neonatal origin, which accumulate in the regional lymphoid tissues and inhibit autoimmune disease [20]. Although they cannot fully prevent development of autoimmune diseases, the autoimmune diseases are dramatically enhanced by in vivo depletion of Treg cells in these mice. On the other hand, the role of thymic ablation in allogeneic immunity largely unknown, although we could speculate about the poor clinical consequences of allo-HSCT recipients with delayed thymopoiesis in recipients with reduced pre-transplant thymic function and in patients suffering from acute or chronic GVHD [21–24]. There has been only a single case report of thymectomy followed by allo-HSCT for a patient with systemic lupus erythematosus, thymoma, and pure red cell aplasia [25]. Although this patient achieved clinical remission of red cell aplasia and lupus, autoantibodies did not disappear after allo-HSCT. It has also been reported that a murine model showed an extrathymic de novo generation of donor-derived T cells against murine cytomegalovirus, although these cells were numerically and functionally reduced compared with thymus-derived T cells [26, 27]. These facts and the existence of naïve Treg cells and Tcon cells in our present study may together suggest that some T cells derived independent of the thymus contribute to immune reconstitution after allo-HSCT. In fact, our thymectomized patient, who had a 100% donor-derived CD3+ cell population including Treg cells, has not required systemic steroids for cGVHD without relapse of leukemia.

There are two types of Treg cells, thymus-derived natural Treg cells, and peripherally generated adaptive Treg cells (iTreg cells) [6–8]. Tcon cells can be converted to Foxp3+ iTreg cells, under the influence of IL-2, but not thymus. In addition, alloantigens can induce Tregs via a thymus-independent pathway in mice [9]. Successful allo-HSCT in our thymectomized patient offers strong evidence that peripherally derived adoptive Treg cells can proliferate, a phenomenon also supported by observation of Treg cells in elderly allo-HSCT recipients in the present study and another previously reported thymectomized patient [25]. This might also partly explain the efficacy of low-dose IL-2 administration [28], and support adoptive Treg cell infusion [29], although the functional difference of Treg cells among young, old and/or thymectomized patients remain unclear. So far, there have been not any specific markers to distinguish natural Treg cells and iTreg cells, but naïve Treg cells observed in our thymectomized patient were considered to include iTreg cells. Indeed, it has been reported that Foxp3+ iTreg cells showed the characteristics of T-cell receptor repertoire of naïve Tcon cells [6]. The role of iTreg cells, in addition to strategies aiming thymic restoration [5, 13, 30], in allo-HSCT should be further investigated.

## Conclusions

Even with pre-transplant thymectomy or older age, both naïve and effector Treg cells certainly proliferated in allo-HSCT recipients. This finding suggests that Treg cells, as well as Tcon cells, can arise peripherally to modulate allogeneic immune responses in humans.

## Methods

### Patients and controls

Twenty-two patients who underwent allo-HSCT from 2008 through 2014, and survived without relapse of hematologic diseases at least 300 days after transplant, were included in this study. In 10 of 22 patients, partial results were previously reported [11]. Median age was 38.5 years with 8 “old recipients” 50 years or older. We compared these old recipients with young recipients (<50 years) to estimate the contribution of age-related thymic involution to the reconstitution of T cells in allo-HSCT. One patient, a 59-year-old female with acute lymphoblastic leukemia, had received total thymectomy to treat invasive thymoma 5 months prior to allo-HSCT.

Patients received tacrolimus and methotrexate as prophylaxis for GVHD, except three who received cyclosporine and methotrexate. All patients received standard supportive treatments including use of laminar airflow units meeting allo-HSCT standards during myelosuppression, prophylaxis of viral infection with acyclovir, administration of granulocyte colony-stimulating factor, and blood transfusion, for which thresholds of 8 g/dL hemoglobin and 20 × 10^9^/L platelets were generally used for packed irradiated red cells and irradiated apheresis platelet concentrates, respectively. Incidences of cGVHD [31] were first diagnosed by standard criteria. Among patients with any cGVHD, we deemed clinically significant cGVHD as that requiring systemic steroid therapy with no less than 30 mg/day of prednisolone, as described elsewhere [14].

This study was approved by Ethics Review Board of Fukushima Medical University, which is guided by local policy, national law, and the Declaration of Helsinki. All investigations were performed after properly documented informed consent. Information and records of patients were anonymized and de-identified prior to analysis.

### Samples

Peripheral blood samples were collected for flow cytometry of Treg cells and Tcon cells at 1 month and/or 1 year after allo-HSCT as described previously [11]. In later evaluation at 1 year after allo-HSCT, we also evaluated naïve and effector cell fractions of each cell type as recently described by Sugiyama et al. [12]. Samples obtained from six age-matched healthy volunteers were used as controls.

### Cell separation, flow cytometry, and chimerism analysis

Peripheral blood mononuclear cells were isolated using Ficoll-Hypaque (Lymphoprep; Axis-shield, Oslo, Norway) and suspended in phosphate-buffered saline containing 0.5% bovine serum albumin (Wako, Osaka, Japan). Anti-CD4 (OKT4; BioLegend, San Diego, CA, USA), anti-CD25 (B1.49.9; Beckman Coulter, Brea, CA, USA), anti-CD45RA (HI100; ALPCO Diagnostics, Salem, NH, USA) and/or anti-FOXP3 (PCH101; eBioscience, San Diego, CA, USA) were used to label PB MNCs after treatment with fixation Permeabilization Buffer (eBioscience). Non-specific control antibodies of the same subclasses were used as negative controls. These PB MNCs were counted by flow cytometry using a FACSCanto II (BD Biosciences), and the data were analyzed with Flow Jo software (FLOW JO LLC, Ashland, OR, USA). For chimerism analysis of CD3+ cells from the thymectomized patient, cells were sorted using EazySep T cell Enrichment Kit (StemCell Technologies, Vancouver, BC, Canada) according to the manufacturer’s protocol. The purity of CD3+ cells, evaluated by flow cytometry, was over 99% in each sample.

### Chimerism analysis

Chimerism of PB MNCs, sorted CD3+ cells, and BM MNCs was determined by short tandem repeat polymerase chain reaction [32] for sex-matched transplants, or fluorescence in situ hybridization for sex chromosomes for sex-mismatched transplants.

### Statistical analysis

To compare categorical variables, the Chi square test was used. The comparison of continuous variables between two groups was made using the unpaired *Student’s* t-test. Correlation coefficient was calculated to determine the correlation between two parameters. All data are shown as mean ± standard deviation unless otherwise specified. All *P* values are two-sided, with *P* < 0.05 considered significant.

## Abbreviations

Allo-HSCT: allogeneic hematopoietic stem cell transplantation
BM: bone marrow
BMT: BM transplantation
PB: peripheral blood
PBSCT: PB stem cell transplantation
GVHD: graft-versus-host disease
aGVHD: acute GVHD
cGVHD: chronic GVHD
Treg cells: regulatory T cells
FOXP3: fork-head box P3
Tcon cells: conventional T cells
iTreg cells: induced Treg cells
MNCs: mononuclear cells
